# Lemierre Syndrome: A Diagnosis behind the Veil

**DOI:** 10.1155/2023/2273954

**Published:** 2023-04-18

**Authors:** Pak-Ho Au, Kelechi Nwabara, Nanuli Gvazava, Shannon Ejiofor, Ghulam Ghous

**Affiliations:** ^1^University Hospital, University of Missouri, Columbia, Missouri, USA; ^2^Ellis Fischel Cancer Center, University of Missouri, Columbia, Missouri, USA

## Abstract

Lemierre syndrome (LS) is a rare, serious infection that is often misdiagnosed, as it frequently mimics common upper respiratory infections. It is even rarer for LS to be preceded by a viral infection. We share a case of LS in a young man who presented to the Emergency Department with COVID-19 viral infection followed by a subsequent LS diagnosis. The patient's condition initially worsened despite treatments for COVID-19 and was subsequently started on broad-spectrum antibiotics. He was then diagnosed with LS after blood cultures grew *Fusobacterium necrophorum*, and antibiotics were adjusted accordingly, resulting in improvement of symptoms. Even though LS is often recognized as a sequela of bacterial pharyngitis, preceding viral infections, including COVID-19, might be a risk factor that contributes to the development of LS.

## 1. Background

Lemierre syndrome (LS) is a relatively unknown condition, indeed sometimes referred to as “the forgotten disease” [1]. First, described by the French physician Andre Lemierre in 1936, LS is a postpharyngitis infection complicated by jugular vein thrombophlebitis, septicemia, and metastatic septic emboli [1–6]. These emboli travel to distance organs, especially the lungs causing further dissemination of disease [1, 2]. The most common bacterium involved is *Fusobacterium necrophorum*, an anaerobic Gram-negative bacillus that is part of normal flora [1, 3, 4]. Circumstances conducive to anaerobic growth allow penetration of the bacteria to the surrounding tissue including the peritonsillar veins and jugular veins [7]. Venous stasis, inflammation, and edema of the veins lead to septic thrombosis, followed by the release of septic emboli [3]. LS has also been seen with *Fusobacterium nucleatum*, *Streptococci*, *Staphylococci*, and *Klebsiella pneumoniae* [1–3].

As LS is not common, it is a diagnosis frequently missed. Data collected between 1998 and 2001 led to an estimated incidence of 3 to 6 cases per million persons per year [2]. Patients are usually young adults who presented with a sore throat that is often misdiagnosed as a viral upper respiratory infection once screening tests for infectious mononucleosis and group A streptococcus infection return negative [1, 4].

In even rarer circumstances, a patient's course may start with a viral infection which makes way for a superimposed bacterial infection and LS [7–11]. In this case, we discuss a young patient complaining of a sore throat, initially diagnosed with SARS-CoV-2 viral infection whose course quickly progressed to LS. Such delay in treatment could have led to detrimental consequences.

## 2. Case Summary

A 20-year-old male with no significant past medical history presented to the emergency department complaining of sore throat and odynophagia for 2 days. He denied any fevers, chills, cough, dyspnea, chest pain, rashes, or headaches. Vital signs were as followed: blood pressure (BP) of 149/76 mmHg with a mean arterial pressure (MAP) of 76 mmHg, temperature (T) of 37.8 Celsius (C), heart rate (HR) of 114 beats per minute (bpm), and oxygen saturation (SpO_2_) of 96% in room air. On examination, his neck was supple and his oral mucosa was moist. There was no uvular deviation or asymmetry of tonsils. The patient did have mild bilateral tonsillar swelling and erythema, mild trismus, tenderness to palpation over the right inferior jaw, and palpable anterior cervical lymphadenopathy. A rapid strep test was negative. SARS-CoV-2 (COVID-19) was not tested at this visit though the patient endorsed exposure from his roommate who was tested positive few days earlier. No chest x-ray (CXR) was obtained. The patient was given one dose of dexamethasone 10 mg. He had one episode of emesis in the Emergency Department after which his symptoms improved as he was now able to speak clearly and swallow his saliva. The patient was diagnosed with viral pharyngitis and discharged from the Emergency Department.

**Figure 1:**
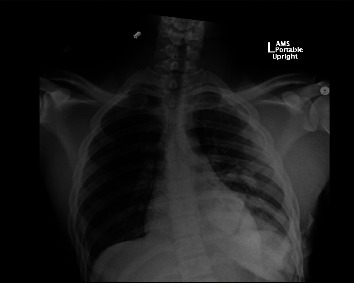
Chest x-ray obtained during the emergency department visit 2. No acute cardiopulmonary process found.

The patient returned to the Emergency Department 13 days later complaining of fevers, chills, vomiting, loss of appetite, nonbloody diarrhea, and fatigue for 3 days. He also reported an episode of “whole body shaking” for 2 minutes witnessed by his roommate. He denied any bowel/bladder incontinence or biting of tongue during this episode and denied a history of seizures. He also denied nasal congestion, rhinorrhea, cough, shortness of breath, chest pain, rash, or dysuria. Vitals at presentation were as follows: BP of 141/80 mmHg with a MAP of 80 mmHg, T of 38.1 C, HR of 137 bpm, and SpO_2_ of 96% on RA. The physical exam was notable for lethargy and dry oral mucosa. No adventitious lung sounds, focal neurological deficits, jaundice, abdominal tenderness, or rashes were noted. Laboratory investigations can be found in Table 1 as Emergency Department Visit 2. He was tested positive for COVID-19 antibodies IgG and IgM. CXR showed no acute pulmonary findings (Figure 1). Blood cultures were obtained. Despite fluid administration, the patient became hypotensive 88/53 mmHg with a MAP of 64 mmHg. Given his fever and physical exam as well as his laboratory result including his positive COVID-19 antibodies and elevated inflammatory markers, the patient met the criteria of multisystem inflammatory syndrome. He was admitted to the stepdown unit, and intravenous immunoglobin G was started (2 g/kg). Cardiology was consulted, and bedside ultrasound showed normal systolic function with normal wall motion and no evidence of effusion. Despite fluid resuscitation, the patient's blood pressure dropped again to 83/48 mmHg with a MAP of 58 mmHg, and he was transferred to the medical intensive care unit (MICU) for further management on day 2.

**Figure 2:**
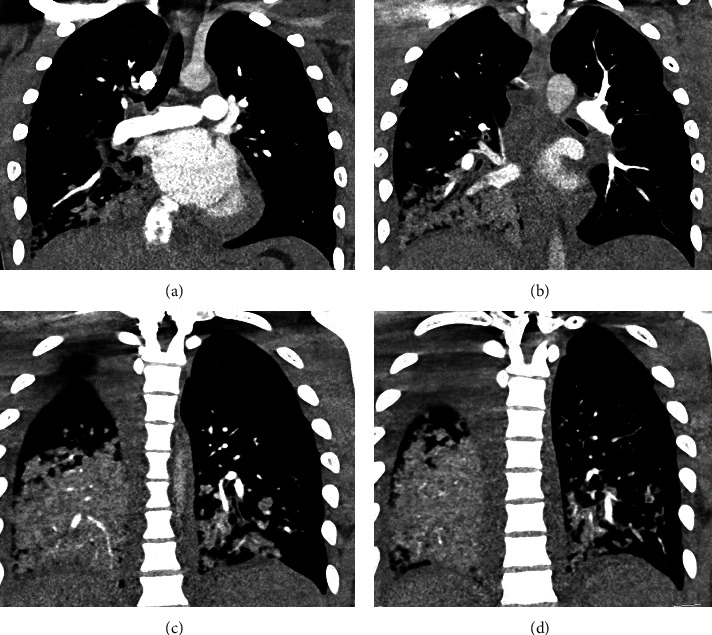
(a–d): Computed tomography angiograms depicting extensive fulminant multifocal bacterial pneumonia with small-moderate bilateral pleural effusions (note the normal appearance of contrast mixing with unopacified inflowing blood in the superior vena cava).

In the MICU, the patient received an additional 2 L of fluids and was started on intravenous ceftriaxone and vancomycin. No pressors were deemed necessary, and he was downgraded to step down unit the same night. However, the patient's respiratory status started to decline rapidly, as he went from saturating 93% SpO_2_ in room air to requiring high flow oxygen (80% FiO_2_ at 40 L/minute). Computed tomography angiogram (CTA) chest excluded pulmonary embolism, but the imaging was evident of multifocal lower lobe opacities with a rapid onset consolidative pattern concerning for fulminant multifocal bacterial pneumonia superimposed on COVID-19 pneumonia (Figures 2(a)–2(d)). On day 3, the patient's blood culture from the day of admission grew *Fusobacterium necrophorum*. His presentation was therefore suspected to be secondary to LS. Infectious disease specialists were consulted who recommended IV ceftriaxone to be continued and IV vancomycin to be discontinued. Initiation of IV metronidazole was also recommended. Neck CTA showed mild narrowing of the internal jugular veins at the level of carotid bifurcation bilaterally, but there was no evidence of external pressure or thrombosis of the internal jugular veins. No anticoagulating agents were ever initiated. The patient's oxygenation status continued to improve, and by day 5, he returned to room air with an SpO_2_ of 97%. The following day, he was discharged with a 4-week course of oral metronidazole 500 mg every 8 hours. The patient did not attend planned follow-up visits with the ID specialists, primary care physicians, and vascular surgery.

## 3. Discussion

LS is a postpharyngitis infection with an estimated mortality rate between 4% and 18% [12].Its relatively high mortality rate is likely contributed by delay in diagnosis as its early-stage manifestations of sore throat, fever, and neck pain/swelling are identical to typical viral and bacterial pharyngitis such as Group A streptococcus, Epstein–Barr virus, and Neisseria gonorrhoeae. These similarities consequently make early detection of LS extremely difficult in practice. As time progresses, further complications of LS occur, including septic embolization to organs causing empyemas, bacterial pneumonia, liver failure, soft tissue and skin infections, brain abscesses, and meningitis. These rare and unusual presentations are rather specific and would make a diagnosis of LS easier, but they only occur late in the course of the disease.

Nonetheless, because the disease is rare, LS remains challenging to diagnose, which may delay effective treatment. In our case specifically, the patient's initial presentation was confounded by concurrent SARS-CoV-2 infection. LS was therefore not considered as a differential diagnosis until positive blood cultures of *Fusobacterium necrophorum* emerged. In this case, broad-spectrum antibiotics were initiated relatively early, possibly contributing to the favorable outcome. While CT imaging can detect a thrombosis of the internal jugular vein, this finding may not be sensitive, and indeed the CT scan did not show a thrombus in our case.

The oral cavity can be exposed to as many as 1,000 microbial species [13]. These include viruses such as herpes virus groups, Zika virus, human immunodeficiency virus as well as bacteria, especially anaerobes such as streptococci, actinomyces species, and *Fusobacterium necrophorum* [14]. The epithelial cells of the oral cavity have the ability to prevent these pathogens from causing illnesses [14]. However, when this primary immunity falters, these microbes can have destructive effects.

The Covid-19 virus has been suspected to use the angiotensin-converting enzyme 2 (ACE2) membrane receptor as an entryway into cells [14, 15]. ACE2 receptors are found throughout the body including oropharyngeal mucosal cells [15]. In one review of Covid-19 patients, the most commonly involved areas of the oral cavity involved the tongue, palate, and labial mucosa; however, 5% of patients had symptoms originating from the oropharynx or tonsils [14, 16]. Though the patient in our case report did not complain of the more common oral symptoms of Covid-19 infection such as dysgeusia [17], he did endorse the vague complaint of a sore throat. The COVID-19 virus may have contributed in the destruction of the lining of the patient's oropharynx through the ACE2 receptors. Furthermore, disruption in blood vessel arrangement may have led to a suitable environment for *F. necrophorum* to grow, as histological analysis of COVID-19 skin lesions has shown changes in vasculature [14]. These histological alterations could eventually predispose patient to LS. While it has been suggested that other viruses cause mucosal damage of the oropharynx triggering the pathogenesis to LS indirectly, there is also hypothesis that viruses can induce thrombophlebitis independently of bacteria as both viruses can elicit a prothrombotic environment directly by mechanisms such as inducing the expression of the tissue factor on monocytes and endothelial cells [2, 45]. Per review, there are three cases in which the patient was diagnosed with influenza and two with Epstein–Barr virus prior to their LS diagnosis [7–11]. According to our search of literature, there has not been any report regarding concurrent COVID-19 infection and LS.

Treatment for LS usually involves prolonged antimicrobial therapy. Depending on clinical circumstances, procedures such as peritonsillar/pharyngeal abscess drainage or chest tube placement for empyema are sometimes indicated to further control sites of infection. Currently, there is no established guideline regarding the selection of antibiotics or duration of therapy for LS treatment. However, when LS is suspected, empiric antibiotics such as meropenem, imipenem or piperacillin-tazobactam should be considered. For LS caused by *F. necrophorum* specifically, metronidazole and ceftriaxone together have been shown to be effective. Such a combination is suggested over metronidazole or ceftriaxone monotherapy due to the frequent coinfection with other bacteria from the oral flora and suspected clinical resistance to penicillin despite in vitro reactivity [6, 17]. A number of case reports described a lack of response to penicillin-based antibiotics alone [19, 20], but there is no systematic documentation regarding overall antibiotic resistance in LS. Clindamycin is known to be an alternative to metronidazole, but monotherapy with clindamycin has been described as ineffective in sporadic case reports. Also, due to its lower bactericidal effect and relatively less efficient tissue penetration in in vitro studies, clindamycin is considered to be a less desirable option than metronidazole [18]. Regarding the duration of antibiotics, Riordan, 2007, referenced an estimate of 9 to 84 days with an average of 42 days [2]. We have analyzed 26 cases of LS from 2016 to 2021, and the average antibiotics duration was 5.3 weeks (Table 2) [4, 17, 21–39, 42–44]. In clinical practice, due to the lack of an established guideline, the duration and selection of antibiotics highly varied based on clinical responses in patients.

The use of anticoagulation in LS remains a controversial topic. Use of anticoagulation in different case reports and reviews has been variable. There has been no definitive controlled study that shows the benefit or harm of the use of anticoagulation in LS [2, 6]. However, some reports suggest the use of anticoagulation in preventing the further propagation of septic embolic events from an IJV thrombosis, even though overall, the suggestion remains anecdotal [40]. In a follow-up study in 2014, the authors described how it remains unclear if anticoagulation will reduce complications and improve outcomes in LS but also stated that anticoagulation appears to be relatively safe with no attributed bleeding complications. Consequently, the authors suggested that anticoagulation should be considered in high-risk patients with extensive IJV thrombosis or extension despite antimicrobial therapy [41]. This perspective is also reflected in the only retro-perspective study available that studied about 700 cases of LS from 2000 to 2017 and concluded that there are no disease-specific elements of concern regarding the safety of anticoagulation in LS [12]. From our analysis of 26 LS cases from 2016 to 2021, anticoagulation was used in 76% of cases and the average duration was 9.8 weeks (ranging from 1 week to 16 weeks; Table 2 [4, 17, 21–38, 41–44]. In our case, due to the absence of thrombophlebitis/IJV thrombosis, no therapeutic anticoagulation was considered.

## 4. Brief Conclusion

LS is a rare but very serious complication of pharyngitis which makes early detection and treatment challenging. It can have insidious onset and rapidly lead to life-threatening complications. Clinicians should be vigilant and suspect LS when symptoms of pharyngitis do not improve over the course of few days, and systemic signs develop after a pharyngeal infection. Detection of distant focus of infection in the setting of pharyngitis should also raise suspicion of LS as septic emboli commonly complicate the course of this disease. Detection of a viral respiratory pathogen should not preclude further workup and in fact may be associated with an increased risk of LS. COVID-19 itself can have systemic manifestations, which may confound and mask another life-threatening diagnosis such as LS. It is also possible that COVID-19 itself predisposes patients to bacterial superinfection via disrupting and altering mucous membrane barrier, which in turn increases the risk of LS [45].

## Figures and Tables

**Table 1:** Pertinent laboratory investigations during second ED visit and upon admission to the MICU.

	Units	ED visit 2	MICU admission	Reference interval
White blood cells	/L	18.78 × 10(9)	11.69 × 10(9)	3.50–10.50/L
Red blood cells	/L	4.35 × 10(12)	4.36 × 10(12)	4.32–5.72/L
Hemoglobin	g/dL	13.3	12.9	13.5–17.5 g/dL
Hematocrit	%	38.3	38.2%	38.8–50.0%)
Platelets	/L	128 × 10(9)	114 × 10(9)	150–450/L
Sodium	mmol/L	139	136	136–145 mmol/L
Potassium	mmol/L	4.2	3.3	3.5–5.1 mmol/L
Chloride	mmol/L	100	101	98–107 mmol/L
Bicarbonate	mmol/L	26	24	22–29 mmol/L
Blood urea nitrogen	mg/dL	16	19	6–20 mmol/L
Creatinine, standardized	mg/dL	1.14	1.11	0.70–1.20 mg/dL
Glucose	mg/dL	142	150	70–139 mg/dL
C-reactive protein	mg/dL	23.74		0–0.05 ng/mL
Procalcitonin	ng/mL	31.90		0–0.05 ng/mL
Ferritin	ng/mL	628		30–400 ng/mL
Lactic acid	Mmol/L		3.2	0.5–2.2 mmol/L
Human immunodeficiency virus 1,2 antigen antibody		Nonreactive		Nonreactive
Monospot		Negative	329	Negative
Influenza A and B rapid point of care		Negative	0.28	Negative
SARS-CoV-2 by nucleic acid amplification test polymerase chain reaction		Negative		Negative
SARS-CoV-2 Ab, IgG, and IgM		Reactive		Nonreactive

**Table 2:** Duration of therapy and anticoagulation use in Lemierre syndrome case reports from 2016 to 2021.

	Author, year	Antibiotics used	Length of antibiotic course	Anticoagulation/Antiplatelet given	Length of anticoagulation course	Source
1	Laurencet, Marie-Eva et al., 2020	Amoxicillin-clavulanate + clindamycin	6 weeks	Acenocoumarol	12 weeks	[21]
2	Goan, Hannah et al., 2021	Meropenem + vancomycin	N/A	Apixaban + clopidogrel	N/A	[22]
3	Lee, Seung Eun et al., 2021	Amoxicillin-clavulanate + clindamycin	6 weeks	Rivaroxaban	12 weeks	[23]
4	Latif, Azka et al., 2021	Vancomycin + pipera cillin/tazobactam ⟶ vancomycin + doxycycline + meropenem	12 weeks	Apixaban	Not specified	[24]
5	Lanfear, Allison et al., 2020	Ceftriaxone and metronidazole	6 weeks	N/A	N/A	[25]
6	Zhao, Andrew, et al., 2017	Piperacillin/tazobactam + IV vancomycin ⟶ ceftriaxone + metronidazole	N/A	N/A	N/A	[26]
7	Santos, Fabio Videira et al., 2020	Ceftriaxone and metronidazole	2 weeks	Enoxaparin	12 weeks	[27]
8	Jaber, Tariq et al., 2021	Ampicillin-sulbactam + 1 dose of gentamicin ⟶ amoxicillin-clavulanic	6 weeks	N/A	N/A	[4]
9	Jaber, Tariq et al., 2021	Ceftriaxone + azithromycin ⟶ piperacillin-tazobactam and levofloxacin ⟶ piperacillin-tazobactam and clindamycin ⟶ amoxicillin-clavulanate	N/A	Heparin ⟶ warfarin	14 weeks	[4]
10	Jaber, Tariq et al., 2021	Cefepime and metronidazole ⟶ ampicillin-sulbactam ⟶ amoxicillin-clavulanate	6 weeks	Apixaban	12 weeks	[4]
11	Jaber, Tariq et al, 2021	Meropenem and vancomycin ⟶ ampicillin-sulbactam	6 weeks	N/A	N/A	[4]
12	Costa, Fatima et al., 2021	Ceftriaxone, azithromycin ⟶ ? maybe kept at same medication	3 weeks	Heparin ⟶ rivaroxaban	16 weeks	[28]
13	Karn, Michele et al., 2020	Clindamycin, vancomycin, ceftriaxone, and ampicillin/sulbactam ⟶ meropenem	5 weeks	Enoxaparin	5 weeks	[29]
14	Alves, Sergio et al., 2019	Amoxicillin/clavulanate + metronidazole	6 weeks	Enoxaparin	14 weeks	[30]
15	Ngu, Vincent et al., 2018	Amoxicillin/clavulanate ⟶ amoxicillin/clavulanate + metronidazole	2 weeks	Not specified	2 weeks	[31]
16	Dasari, Suhas et al., 2020	IV ceftriaxone and oral metronidazole	4 weeks	Warfarin	12 weeks	[32]
17	Mellor, Thomas et al., 2017	Piperacillin/tazobactam ⟶ ertapenem	4 weeks	N/A	N/A	[33]
18	Gohal, Satan et al. 2021	Piperacillin-tazobactam and clindamycin	6 weeks	Enoxaparin	Not specified	[34]
19	Jawad et al., 2018	Amoxicillin/clavulanate + clarithromycin ⟶ piperacillin-tazobactam + metronidazole ⟶ amoxicillin/clavulanate	6 weeks	Low molecular weight heparin ⟶ rivaroxaban	12 weeks	[35]
20	Gonzalez et al., 2019	Vancomycin and piperacillin/tazobactam ⟶ vancomycin	4 weeks	IV heparin ⟶ oral apixaban	10 days	[36]
21	Garcia et al., 2017	i.v, cefepime, and metronidazole ⟶ IV metronidazole ⟶ amoxicillin‐clavulanate	6 weeks	IV heparin ⟶ warfarin	Not specified	[37]
22	Barguil et al., 2021	Ceftriaxone, metronidazole, and linezolid ⟶ amoxicillin-clavulanic acid	4 weeks	Enoxaparin	4 weeks	[38]
23	Sattar et al., 2020	Meropenem ⟶ piperacillin/tazobactam ⟶ clindamycin	3 weeks	Heparin ⟶ warfarin	Not specified	[39]
24	Howley et al., 2020	Ceftriaxone, vancomycin, and metronidazole ⟶ intravenous ceftriaxone and oral metronidazole	12 weeks	Enoxaparin	12 weeks	[40]
25	Man, Man-Yee et al., 2018	Doxycycline, clindamycin, and vancomycin ⟶ meropenem + doxycycline ⟶ amoxicillin-clavulanate	6 weeks	N/A	N/A	[41]
26	De Oliveira, Rodrigo et al., 2018	Azithromycin, clindamycin, and ceftriaxone ⟶ clindamycin, and ceftriaxone	10 days	N/A	N/A	[12]

## Data Availability

The data used to support the findings of this study are available from the corresponding author upon request.
